# A qualitative exploration of feedback experience among final-year physiotherapy students using activity theory

**DOI:** 10.1186/s12909-025-06635-8

**Published:** 2025-01-20

**Authors:** Alison Lupton-Smith, Nicoline Herman, Anna Schmutz

**Affiliations:** 1 Division of Physiotherapy, Stellenbosch University, Cape Town, South Africa; 2 Department of Health Professions Education, Stellenbosch University, Cape Town, South Africa; 3 Centre for Teaching and Learning, Stellenbosch University, Cape Town, South Africa

**Keywords:** Feedback, Student agency, Feedback literacy, Activity theory, Cultural-historical activity theory

## Abstract

**Background:**

Feedback is an important part of learning, however, it often does not have the desired effect. Much work has been done exploring students’ engagement with feedback and factors which may impact engagement. Mutual understanding of feedback and feedback practice on the part of the student and educator is essential.

**Methods:**

This study explored the perceptions of feedback of final-year physiotherapy students at Stellenbosch University (South Africa). Twelve semi-structured interviews were conducted to generate data. Cultural-historical activity theory was used as an analytic lens in the data analysis.

**Results:**

Students understood feedback to be a continuous, bidirectional conversation in which they were actively involved. Students recognised their agency in feedback practice. Despite their understanding, their agency was often undermined by factors such as the environment, context and most notably the perceived positioning and relationships between students and educators. Educators who were perceived as credible and created a safe psychosocial space had a positive influence on the students’ perception of feedback.

**Conclusions:**

Students’ perceptions of feedback and their engagement was the product of a complex and dynamic interplay of factors. While students recognise their agency, this may be hindered by relationships and the design of feedback in the curriculum. As educators, one must consider how we contribute to this and enable students to activate and use their agency in feedback practice.

**Supplementary Information:**

The online version contains supplementary material available at 10.1186/s12909-025-06635-8.

## Introduction

Feedback is undoubtedly an essential aspect of learning [1–3]. Re-conceptualisations of feedback speak to a shift from transmission-focused (or educator-centred feedback) to student-centred feedback [2, 4]. The student-centred model proposed by Boud and Molloy (2013), recognises the role and agency of the student as central to feedback as a process [2, 5, 6]. Despite recognising the centrality and agency of the students to feedback practice and the increase in publications on how to improve the feedback practice, the voice and agency of the students in effective feedback practice has, until recently, been somewhat muted in the literature [3, 7, 8].

Ossenberg et al. (2019) highlight the gap in the literature around student engagement in feedback and therefore, a potential gap in understanding feedback effectiveness. This lack of engagement may be a result of a disparity between the student and educator about the purpose of feedback and the roles and responsibilities of the individuals involved in the feedback process [9, 10]. On the part of the student, a comprehensive understanding of the purpose of feedback could better facilitate their role and engagement in and with feedback [5, 6, 11, 12]. Consequently, poor understanding of the purpose of feedback on the part of the students can severely hamper the effectiveness of feedback practice and subsequent lifelong learning and development of the student [5, 13, 14]. Furthermore, a lack of cohesion between the dimensions proposed by Yang and Carless (2013) may further impact student engagement despite students’ understanding [9].

In keeping with the re-conceptualisation of feedback, Yang and Carless (2013) propose a feedback framework which encompasses both the cognitivist and social constructivist approaches to feedback [9]. They propose that for feedback to be effective, three interrelated dimensions exist, namely the cognitive, social-affective and structural dimensions [9]. This framework draws attention to the importance of self-regulation on the part of the student [15]; the relational aspect of feedback and the role of emotions, identity and power dynamics in feedback practice [15–17]; and lastly, the importance of practicalities of feedback practice such as policies, time limitations, limited resources etc [2, 6, 9, 18].

Recognising the importance of a mutual understanding of feedback and the roles and responsibilities, feedback literacies have been developed for students and educators [5, 12, 19, 20]. The majority of this work is focused on student feedback literacy and the development thereof, however, is it increasingly being recognised that feedback does not exist in isolation, but is the result of a dynamic interplay between people, the environment and contextual factors [21–25].

While the aforementioned literacies provide a foundation, they have been critiqued for not taking into account the complexity of feedback practice and are largely competency-based (for students in particular) [26]. Assuming a less cognitivist approach to these literacies, it has been postulated that literacies are more fluid and emerge in feedback practice depending on the people, context and environment [25, 26]. Furthermore, these literacies extend beyond just people and one needs to consider the complex and dynamic interplay within and between the people, environment and context [27].

Insight into students’ understanding and experience of feedback, while recognising the complexity of feedback practice, may help identify areas and future interventions for improving feedback practice and in turn better equip students to engage with and grow from feedback. Therefore, this study aimed to explore final year students’ understanding, experience, and consequent perception of feedback in an undergraduate physiotherapy programme.

## Materials and methods

A qualitative exploratory study within an interpretivist paradigm was conducted. All the 2022 final year Bachelor of Science (Physiotherapy) students at Stellenbosch University (SU) (Cape Town, South Africa) were invited to participate in the study (*n* = 45; *n* = 6 (13%) male; *n* = 39 (87%) female). In this four-year bachelor’s degree programme, students have predominately class-based activities in the first two years. In these years, students are primarily exposed to individual written and verbal feedback concerning assessment (both theory and practical). In addition, more general class-based feedback may also take place. In the third and fourth years of study, students begin with clinical training which takes place in a variety of health care settings. In the fourth year of the programme, most of the year is spent in clinical training. During clinical training, students are responsible for the physiotherapy management of clients under supervision. In these years, students engage with feedback from multiple sources such as clinicians, clinical educators (employed by SU), peers, patients and academic staff. There is currently no formal feedback training for students in the curriculum, however, informal feedback training may take place. While staff development concerning feedback is not a requirement, all involved with the training of students have access to various staff development workshops run in the Physiotherapy Division and the Faculty of Medicine and Health Sciences as well as in the institution (SU).

This cohort of final-year students was chosen as they had a variety of contexts and opportunities to engage with feedback, in both classroom and clinical settings. The changes to teaching, learning and assessment that occurred as a result of the SARS-CoV-2 pandemic, meant that this cohort of students had experienced both face-to-face and online modes of feedback which further informs the context of this study.

Convenience sampling was used and no exclusion criteria were applied. An email detailing the study was sent to all fourth-year physiotherapy students by the primary researcher (ALS), and all those who responded and provided informed consent were included in the study. Due to limited responses to repeated email invitations from the primary researcher (ALS) and recognising that it was busy time of year and following the completion of assessments, the students may not be checking their emails regularly, snowballing was used as an alternative sampling strategy. *S*nowball sampling was employed whereby the participants reached out to their peers asking them to participate if they were interested [28]. A total of 12 students (26% of the class) were included in the study. Eleven students were female (92%), and none of the students were repeating the year. Students included each had a range of different clinical exposure including sites, clinicians, and clinical educators. No participant withdrew from the study.

### Data generation

Data was generated via semi-structured individual interviews (between 30 and 45 min long). A self-developed interview guide was developed to answer the primary research question (Supp 1). The interview guide was reviewed by all authors and piloted to determine whether any changes were necessary. After piloting the guide was deemed appropriated and data generation was begun. Interviews were conducted by the primary researcher (ALS) either in-person or online via Microsoft Teams depending on participant availability. Given that the primary researcher (ALS) was relatively novice, piloting allowed her to familiarise herself with the process and develop her skills. The recording of the first interview was reviewed by a second researcher (AMS) as a quality control measure. All interviews were audio-recorded and transcribed verbatim by a research assistant. Having done the interviews, ALS read though all the transcripts as a cursory check. Where clarity was needed or tone was maybe important, the ALS went back and listened to the interview for clarity and included changes in the transcripts.

### Data analysis

Data were analysed in two phases. The first was an iterative, inductive and exploratory process following the process described by Braun and Clarke (2021) [29]. The primary author (ALS) immersed themself in the data by checking and re-reading the transcriptions [29–31]. After codes were generated, the codes were confirmed by co-researchers (NH, AMS) [29, 32]. Codes were grouped and mapped. This process was repeated until the data coherently aligned with the research question as recommended by Braun & Clarke (2006, 2021) [29, 30]. The process of constructing, reviewing, refining, mapping was clearly documented. Atlas.ti v.24 (ATLAS.ti Scientific Software Development GmbH, Germany) was used during coding and the categorisation of data. Trustworthiness was further augmented by the co-authors checking the codes, categories and identified themes throughout the process [29, 32, 33]. This process allowed us to immerse and familiarise ourselves with the data. Data were then deductively analysed through the lens of cultural-historical activity theory (CHAT) [33].

Cultural-historical activity theory (CHAT) has its origins in the work of Lev Vygotsky, where he proposes that learning occurs through interactions which are facilitated by cultural tools (such as language) and these tools themselves have over time (history) evolved [34]. This idea was further developed, where the role of motivation and the collective is recognised as fundamental [33, 35]. These later versions, highlight the functioning of an individual within a social (Leont’yev) and contextual system [33].

CHAT considers the network and interplay between activities and role players, which is dynamic and evolving, making it a potentially useful analytic lens for feedback [35]. Given the complex and dynamic interplay among a number of factors in feedback practice, activity theory provides a useful lens for understanding students’ understanding and experiences [2, 9]. Furthermore, activity theory enables the analysis of individual factors contributing to their understanding and experience of feedback, as well as the interplay between these factors, therefore allowing for a student-centred and student-driven approach to feedback to be recognised [2, 36, 37].

**Fig. 1:**
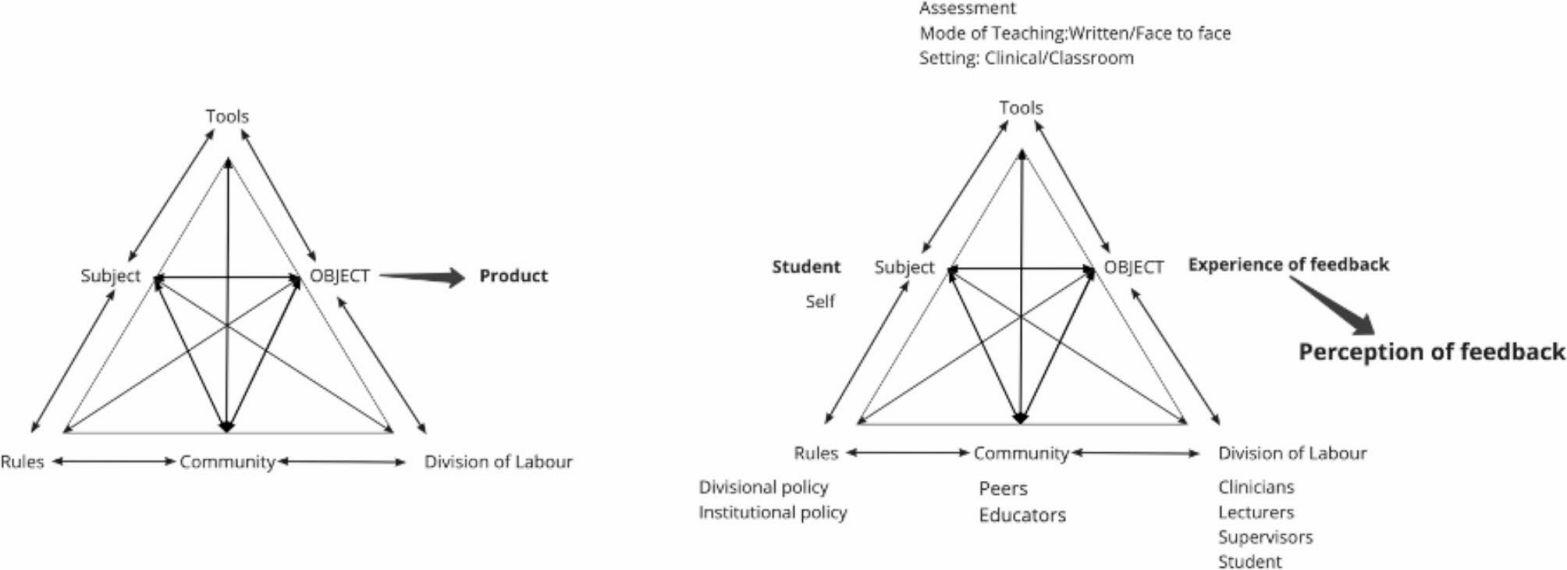
Third generation activity system/triangle (Engeström 2001) (left) and the proposed activity system used in this study (right)

Within activity theory, the ‘activity triangle’ is a visual representation of the various contributors to the activity system and interactions within an activity system (Fig. 1, left triangle). Each activity system has six key entities namely the subject, object, community, rules, division of labour and tools (Fig. 1 left triangle), which through their interaction culminate in the product of the activity. The *subject* is the entity acting on the object or whose perspective is being evaluated. The *object* is the motive of the activity or the ‘problem space’, which is transformed through the enactment of the subject, mediated by *tools*/resources available, on it [34, 38]. The system includes the *rules* (norms/social conventions) governing the subject/system, the role players involved (*community*), and the roles and responsibilities assumed by role players (*division of labour*) [34]. Within the activity system, there may be ‘tensions’ or ‘contingent forces’ which may be historical or cultural in nature [36, 39, 40]. Tensions can exist between entities (e.g. subject-division of labour) or within a single entity (e.g. subject) [36]. These tensions are viewed as spaces of possibility, and it is only when they are brought to light that change is possible [36]. The result of these interactions and tensions within the system ultimately culminates in the product of the activity.

Using the feedback framework proposed by Yang and Carless (2013) as a guide, a proposed activity system for feedback is shown in Fig. 1, the triangle on the right, which was then used in the deductive data analysis [9]. The structural domain was used to identify tools, rules and the division of labour. The cognitive domain informed the subject, division of labour and community, and lastly, the socio-affective domain informed the subject, and community, and was considered when examining the arising tensions. The **Subject** of the proposed feedback activity system is the students. Students have been selected as the subject of this activity system, as they are the ones engaging with and acting upon the feedback that informs their experience [41]. The **Object** of the system is the students’ experience of feedback. **Tools** represent the various teaching, learning and assessment methods. Recognising the relational aspects of feedback, the **Community** represents those students who may interact with educators, peers and patients during feedback. The **Division of Labour** can be categorised by the role/responsibility an individual in the community may assume, such as educator (academic staff and/or clinical educators), student, clinician etc. Lastly, feedback practice may be governed by various **Rules** of the system which include institutional (SU) and divisional policies related to teaching, learning and assessment and the contexts in which the students operate. The **Product** of the feedback activity system is the students’ perception of feedback.

### Data quality

Trustworthiness was augmented by the co-researchers checking the codes and categories throughout the data analysis process [42]. Since we were immersed in the data, our potential influence on data generation and interpretation was recognised. The primary researcher (ALS) believes and understands feedback to be a dynamic and active conversation between the parties involved. Feedback is aimed towards learning and growth and can occur in various ways. Importantly, participating in feedback requires vulnerability by all parties, which in turn requires a relationship. Since the researcher (ALS) was immersed in the data, the potential influence on the data generation and interpretation, based on her beliefs and understanding, was recognised. Throughout the research process, a critical and reflective account of all research processes in the form of a journal was kept [43, 44]. The primary researcher engaged with the co-researchers as a sounding board where there was a concern that her beliefs might be muddying the interpretation of the data. Both co-researchers (AMS and NH) have extensive experience in the field of education at both undergraduate and postgraduate levels. They frequently engage with feedback practice and acknowledge the role their past experience and practice could have on the data interpretation. Regular meetings between researchers contributed to uncover potential assumptions and contribute to a transparent approach to the data analysis, interpretation and findings.

### Ethical considerations

Ethical approval was obtained from the SU Health Research Ethics Committee (S22/03/043), Institutional Governance (SU) and the Division of Physiotherapy before starting the recruiting of participants. The study was conducted in accordance with the Declaration of Helsinki (2013). Written informed consent was obtained from all participants in the study. Since the primary researcher (ALS) was involved in teaching and assessment of the students, there may have been a power dynamic affecting participant’s responses. To mitigate this, participants were given the option to indicate if they were uncomfortable being interviewed by the researcher, and if this was the case, the interviews would be conducted by an independent, trained research assistant. However, no participants objected to being interviewed by the primary researcher (ALS). The interviews also took place after all of their final assessments had been completed which may have helped reduce the power dynamics. Given that new graduates in South Africa are automatically appointed into a job post by the Department of Health, participation in the study did not affect their employment in the following year.

No large language models were used in the generation of this manuscript.

## Results

**Fig. 2:**
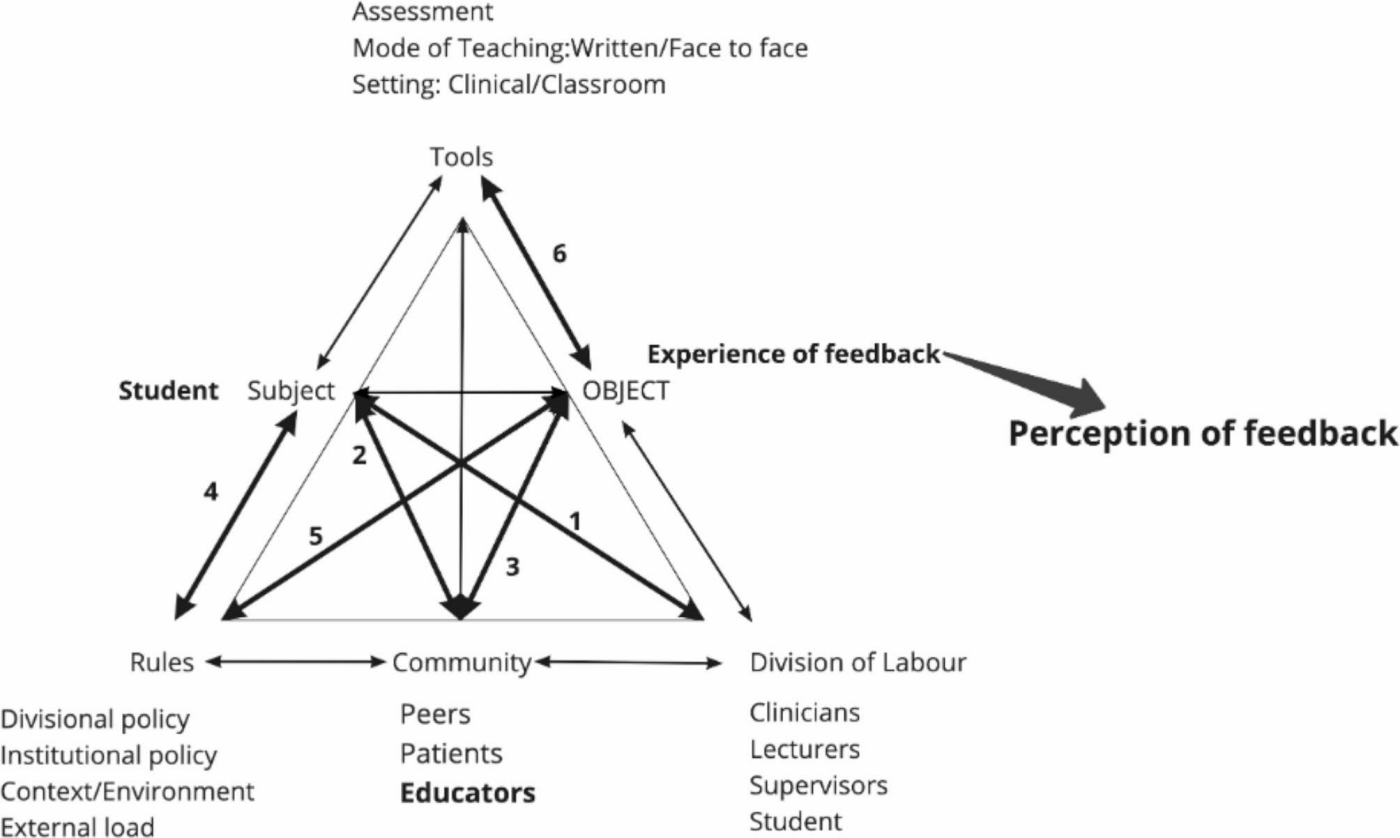
The activity system with tensions between nodes indicated by the bold arrows and numbers. Tensions within nodes are indicated by bolded text

Students entered the ‘activity’ of feedback with some understanding and experience which has been informed by their past experience (historical) and different contexts (including culture). We first explored the students’ understanding of feedback and, hence the stance they took when engaging with feedback and entering the activity system. We extrapolated their stance into the feedback activity system where the various factors which contributed to the students’ experience and consequent perception of feedback were explored. Additional elements that were added to the system were based on the student data shown in Fig. 2. We will highlight and explore the tensions within the activity system from the students’ point of view.

### The students’ stance on entering the system – their understanding

All students seemed to understand the purpose of feedback to be toward their professional and academic development. Some students appreciated how feedback contributed to their personal growth (e.g. in confidence). Students saw feedback as making an important contribution to their learning (‘*So for me*,* feedback is one of the most valuable tools for learning’ P10*), and skills development. This contribution to their learning was achieved through gap identification, meaning making (understanding the why *‘*… *so that it sticks in somewhere in our brains. ’ P3*) and reflection *(‘because internal feedback is more like reflection’ P4)*. Students recognised that feedback was more than a knowledge transaction, but aimed at improving their understanding (*‘Not only an answer but an understanding of the question you asked’ P9*) and required further action on their part (‘*not just something that’s said*,* but something that’s said and can be acted on’ P9*).

Feedback was understood to be a *‘continuous process’ (P10)*, which was an active, bidirectional *‘process instead of just it being one-sided from either only the student part or only from the facilitator’s side.’ (P3)*. This bidirectional process was viewed to be a conversation i.e. *‘not talking at me*,* but like talking with me’ (P3).* The active role and responsibility of students in acting on the feedback was recognised and understood - *‘feedback so whether that’s implementing into the next patient or into the way you study*,* that there’s a responsibility to do something with the feedback.’ (P12)*. Students acknowledged their responsibility to actively seek out feedback (*‘if you’re looking for feedback*,* an opportunity to find it*,* you will find [it]*,* in day-to-day things.’ P1)* and recognised the multisource nature of feedback. Many students also recognised that *‘feedback’s not something that you should just take blindly’ (P9)* because *‘sometimes you get feedback and you don’t completely agree with it*,* but at least reflect on it*,* or get feedback from someone else…’ (P9)*, highlighting the need for reflection on the part of the student.

Several students considered feedback as an opportunity *‘to see through the [assessor’s] eyes’ (P3)* or do what the assessor wanted with a stance to improve in the next assessment. Others, saw it as an opportunity to improve their care of others and recognised that *’feedback is important to build your relationship to effectively care and treat people.’(P1).* Students acknowledged that feedback comes from multiple sources such as educators, peers, hospital staff, patients and marks. While most students valued feedback from a more knowledgeable person, they highlighted the value of peer feedback, ‘*because they’re [peers] kind of experiencing the same thing and also our knowledge or experiences are on the same level…you can also kind of relate be like*,* yes*,* I also felt like this*,* and this is what I did.’(P7).*

Students expressed a definite shift in their appreciation and seeking out of feedback when they started their clinical rotations because, there were now implications for the people they were treating (‘*…doing it for people*,* to serve and help people*,* and that’s the point at the end of the day. Making them a priority. It’s not about the mark*,* or the assessment.’ P1*). Students also recognised that their understanding of feedback had been informed by past experiences *(‘I was very scared of feedback*,* I hated being wrong. I don’t like it when people tell me that I’m wrong*,* especially that first year. But now*,* I mean this year the thing that I valued most was that constant feedback*,* from anyone honestly.’ P10*). Despite past experiences, students reported a shift in their perception of feedback being negative to it being positive and something necessary to improve *‘because there’s the discomfort of just realising: ‘Oh my goodness*,* this is really uncomfortable that I don’t know this’ and like this person is like digging into the fact that I don’t know this. That’s uncomfortable*,* but it’s more like I know there’s something good coming out of this.’ (P5)*. This sitting and grappling with the discomfort was facilitated through reflection.

The affective component of feedback was highlighted by students with almost all of them recognising that feedback elicited emotions. These emotions were either positive or negative. Some students reported that *‘*[emotion] *puts things totally out of context.’ (P2)* which impacted how they then engaged with feedback and their perception of future feedback encounters. We found that some students learnt to deal with the affective component through reflection (*‘I think feelings towards feedback are valuable*,* but I think valuable to like reflect on first before taking any action.’ P1*). Most of the students found that affirmation seemed to help them better deal with the ‘negatives’ (*‘because she said: ‘You know*,* you did well*,* you’re not on the wrong path.’ it helped me to feel more competent to go treat a patient ’ P6*).

### The student experience of feedback – the activity system

The factors interacting in the system and informing the students’ experience of feedback are shown in Fig. 2. We found several tensions in the system between nodes which are indicated in Fig. 2. Additionally, we noted two tensions within nodes, namely within the student node and with the community node, and these overflowed into other tensions.

### Tensions in the division of labour (tension 1)

While students entered feedback engagements with the aforementioned understanding, it was noted that how they positioned or viewed themselves potentially affected how they experienced the feedback. This created tension within themselves. One student verbalised that knowing who they were i.e. their identity, added to their engagement with the feedback and allowed them to navigate the feedback (*‘I think it comes down to like knowing who you are*,* like identity and like being pretty secure in who you are and taking stock of where you’re at…’ P1*).

How students positioned themselves within the feedback engagement, related to their knowledge, seemed to contribute to their affective response to the feedback and consequent engagement, as one student said *‘I think if I don’t know something… I’m going to feel threatened because I don’t know this thing that I’m supposed to know it so then that can also bring up negative emotions.’ (P3)* and another said ‘*sometimes you feel like it’s degrading on your intellect*,* but it’s even then it’s emotional because you’re feeling that you*,* your pride is taking a hit…’ (P8).* The responses from the students to affect, varied where some had active, immediate defensive responses such as the following example*‘…my own reputation. I wanted to know why I didn’t get it right and then I wanted to fight it because I really believed in what I said.’ (P12)*. We noted that others experienced something akin to paralysis and moving beyond the feedback was difficult for them (*‘I think to take feedback negatively would mean to shut down afterwards and respond passively ’ P1*). However, feedback was also seen as an opportunity to grow despite the emotions and discomfort elicited. It appeared that their constructive response was mediated by self-reflection and self-awareness *(‘Like*,* I think that understanding where you’re at is important*,* like a stock take of kind of*,* what do I know*,* and where are some of my gaps.’ P1*) and being able to stand back and separate themselves from the emotions and not taking the feedback personally (*‘… not it’s you as a person being a bad person…but that’s also a learning curve*,* that also takes a while to separate yourself as a person*,* like separate me from the student.’ P10*).

Another tension in the division of labour (Fig. 2 – tension 1) arose with how students positioned themselves in relation to the roles and responsibilities in the feedback engagement. We noted that the students’ experience and engagement with feedback was limited if they placed a high perceived value on the expertise and knowledge of the other person in the feedback engagement (*‘I wasn’t confident in my knowledge*,* because I was so worried about their knowledge’ P5*). While not wanting to be seen as ‘*just* students’, students mentioned the importance of realistic expectations from those engaging in feedback with them and for those engaging to ‘*remember that they are working with students*,* who are not 100% trained professionals yet on that level.’ (P10)*, and that *‘*[students] *don’t know everything*,* we do sometimes struggle. But giving us the grace to improve and helping us.’ (P4)*. The navigation of these expectations, roles and responsibilities, seemed to be influenced by their perception of the person engaging with them (*‘So I think the person*,* if you don’t see them as approachable or gracious and like*,* at least a little bit kind*,* it’ll be difficult because the way they deliver it might be harder than if someone that was a bit more approachable delivered it’ P1*). When some students perceived the role of the educator as that of merely fulfilling a duty, the students’ experience and consequent engagement with the feedback was diminished (‘*And can I see that you want to help me*,* or are you just there to kind of do the job that you paid for’ P11*).

We found that with some students, the perception of power dynamics or hierarchy within the feedback engagement elicited a strong defensive response to feedback (*‘Retaliate… on the lower level of the hierarchy is that you want to fight back.’ P12*). It is possible that these perceived power dynamics may arise from unclear expectations on the part of both the student and educator, for example, one student said *‘You know*,* stick to her rules kind of thing*,* you know what her boundaries are. And or what she expects essentially. And then you’ll not get in trouble kind of thing.’ (P2)*.

If those engaging in feedback with the students did not recognise the active role or agency of the student or saw feedback as a conversation, the implications for student engagement were often negative (*‘I usually am freer in asking a lot of questions when the environment is sort of open for questions. And when it’s a more closed environment*,* where there’s only one person that’s talking*,* then I just tend to come into my shell and I just keep quiet’ P3*). We noted that when those engaging with students were perceived to take a student-centred approach to feedback and when ‘*educators start[ed] with listening to what the student’s want their feedback on*’ *(P9)* the students’ experience was ameliorated.

### Tensions in community (tensions 2 and 3)

We found a tension within the community, especially between educators and clinicians. A perceived discrepancy between the practice of what is taught and what is expected or practised made it difficult for some students to navigate and reconcile feedback (*‘I go to clinical and this clinician… is saying things that like contradict what I’ve learned which is difficult because everyone has their own perspective and their own physio practice’ P11*). Students felt that educators *‘are more into the literature and what the university expects of us’ (P6)* and while students valued the input from clinicians, they may take the clinicians’ feedback *‘with a little bit more caution than the supervisors* [educators]*’(P6)*. For the students, the community member’s credibility in terms of knowledge of literature and an expectation of what students should know mattered.

We found that the students’ view of those in the community, specifically the educators *(‘I think it has a lot to do of like what we see in our like in our lectures*,* like what we learn also.’ P11*), together with the educators’ attitudes and behaviour were important to their feedback experience (tension 2). What educators role modelled, affected how students further engaged with feedback (*‘I think for me like a big part of that is like how you [educators] conduct yourselves around your students and your patients.’ P11*). The behaviour of those engaging with the students was highlighted by students as affecting their responsiveness and experience of feedback. Students frequently referred to an ‘*unspoken safe space*’ *(P12)*, which appeared to be fostered by the behaviour of the educator. Being seen as someone interested in a student as a person (*‘I felt more at home or comfortable*,* with the people building sort of relationship and finding out about you as a person outside of physio’ P2; ‘and also that they are seeing some potential in you.’ P11*) and perceived as someone who listens to students (‘*and someone actually listening. I was also more capable of receiving negative feedback from them’ P6*) were important contributors to this space and the credibility of the educator. Whether or not educators acted on feedback received from students appeared to contribute to the perceived safety of the space and the extent to which students then engaged in feedback (‘*Because when you as a student*,* you quickly learn who the lecturers and people are that don’t really listen to your feedback.’ P9*). This space was further informed by the perceived intentions of the educators and whether they *‘had intentions to teach and to enhance.’* *(P12)* or if it was just a job for them.

Students identified various factors in the feedback engagement that negatively influenced their experience (tension 3). The most common included aspects like *‘the tone* [with] *which the supervisors deliver that feedback. Being shouted at with constructive feedback is still being shouted at…’ (P10)*. Body language was also important to students - ‘*I’ve noticed with the feedback that I’ve received*,* if the body language is negative*,* I get a lot more stressed inside.’ (P5).* While subtle body language was referred to, more obvious cases were mentioned like ‘*they were just sitting there with like their legs crossed and arms crossed*,* lying back in the chair*,* sighing a few times.’ (P10)*. Behaviours like arriving late, being on one’s phone or taking over while observing the student or during the feedback engagement were also reported as negatively impacting the students. The attitudes of those engaging with the students and how they positioned themselves relative to the student was highlighted in that they may *‘Give their feedback… in that way*,* it does more damage*,* but it kind of brings you down more than puts you up.’ (P8)*.

### Tensions with rules (tensions 4 and 5)

We found that students described something akin to an “additional load” which was not necessarily under their control and included it in the rules of the system (Fig. 2). The “additional load” was described by some students as e.g., competing academic demands of the undergraduate programme, the cognitive load (*‘academically I’m currently in a space where I’m constantly being challenged every day…So I’m constantly in a process of reflecting*,* trying to fill my gaps. And it becomes draining to be doing that the whole time*,* even though it’s good*,* you need to like try and find a balance with that.’ P1)* and/or the emotional load of some clinical placements ‘*sometimes there is just a lot going on*,* and you’re feeling very emotional and overwhelmed with other work as well*,* and you’re already not in the best space to be in.’ P6*). From the data, we noted when students experienced this “additional load”, their capacity to engage with and interpret feedback, may have been limited (tension 4) as was noted by a participant *‘so I think that what I’m dealing with at the time could make it difficult to ask for feedback*,* because I’m like*,* like that’s another thing to like ask for*,* and it’s probably going to like create questions in my mind that I might not have the capacity to deal with at the time.’ (P1).*

A few students alluded to how the rules (tension 5), namely divisional policies around feedback, were not always helpful to their experience as the standard period of two weeks for receiving feedback following a written assessment was too long *(‘we did this*,* that’s like a long time ago and I’m not as invested*,* or still in that mindset in a way.’ P7*) and, therefore, not helpful to their learning. Students also noted that while immediate feedback (particularly in the area of clinical skills) was helpful, while others reported the opposite and recognised the need for time to lapse to allow for self-reflection before receiving formal feedback, ‘*because I do really value that self that self-introspection feedback time because that’s also a form of processing. Yeah. Processing and clearing the mind so that you can actually receive the feedback.’ (P12)*.

The clinical environment added an additional layer of complexity to the rules of the system (indicated by context, tension 5). In this setting, students highlighted how potentially uncontrollable factors in the physical environment impacted their experience of feedback. Students found that noisy and busy environments made it difficult to engage with the feedback ‘*if you just standing in the middle of the busy ward*,* it’s really hard to concentrate sometimes. So then you’re not going to take in what’s being said*,* whereas if it’s like in a quiet room that also helps’ (P4).* Furthermore, the presence of other people was noted to potentially make it more challenging to fully engage with the feedback (*‘If someone had to give me feedback that maybe was a bit harder to receive*,* not negative*,* but harder to receive*,* in a setting in front of people… ’ P1*) and students seemed to be able to engage better if there was privacy. The availability of equipment and space for example having chairs to sit on could, for some, also have made a difference in their affective experience (*‘we were standing*,* and it felt like we were in a fight. I’m*,* I’m very scared of conflict so I’d do anything to avoid conflict*,* I don’t like conflict at all. I think maybe had we been sitting I would have felt different.’ P3*).

### Tensions with the tools (tension 6)

Students indicated their preference for face-to-face in-person feedback as this allowed for conversation and clarification on the part of both them and the educators (*‘But I think it’s sometimes better to talk through it because sometimes I say something on the evaluation form and then they give feedback on the evaluation form*,* and I don’t really understand what they mean*,* or they don’t really understand what I meant.’ P6*). Some students noted that while there was a place for written feedback, often this was generic and impersonal. Students wanted to have a conversation to question, understand and aid their meaning-making (*‘I personally think verbal feedback is the best way. I don’t think having five lines on a piece of paper is enough… I also respond better if I can actively talk to you about it*,* if I can ask you: ‘okay*,* but why? Why that instead of this or…’ P11*). It seems that some of the tools used, such as rubrics, did not necessarily facilitate student-centred or enough feedback to aid in students’ understanding. One student found this frustrating and stated that ‘*it’s not really feedback; it’s just reiterating the rubric… that’s not really feedback. I can read the rubric.’ (P11*).

## Discussion

Feedback, as understood by the students in our study, is an active two-way conversation towards one’s personal and professional growth [2, 15, 45]. Furthermore, feedback was seen as a continuous process in keeping with the idea of feedback spirals rather than loops [46]. The view of this continuous spiralling dialogue, as well as the reflection may also speak to students seeing themselves as co-creators of the feedback [18]. However, while students saw their role as active in the process and recognised their agency, tensions arose when the same understanding was not reciprocated by the educators, highlighting the importance of a mutual understanding of feedback practice [47]. Students recognised the role emotions played in their experience and perception of feedback [9]. Recognising the impact of emotions on their feedback engagement, and processing these through reflection contributed to a meaningful feedback experience [7, 48]. Students in our study demonstrated key aspects of feedback literacy [5, 12].

The beginning of clinical practice triggered a greater appreciation for feedback in students, their agency and the motivation to seek out feedback, as they became responsible for the care of others [7]. This shift may speak to the maturity of these students and is in keeping with the principles of adult learning [37, 47, 49]. The concept of agency in feedback has been linked to student identity, confidence and how they see themselves as students [2, 47, 50]. This was evidenced in some of our students who recognised the role of identity and may also account for the tensions which arose within the student node around how some students positioned themselves in the feedback interactions. These tensions in this mature group of students may also speak to the case of design and whether student agency is enabled by educators and environments [2, 5, 6, 10, 18].

Various proposed models and frameworks have highlighted the role context and environment can play on student agency and feedback practice [9, 12, 18, 51]. Student agency, in our case, was enhanced through the need to improve future performance to improve their care of people [18]. However, their agency and consequent experience were also affected by the environment and non-human entities (tools) such as space, time and in some cases, tools like rubrics [18, 22, 25, 52]. These tools often negatively affected the students’ experience with feedback and consequent engagement and perception. Despite the increasing use of technology in teaching, learning, and assessment, students preferred in-person feedback as it better facilitated conversation and allowed them to clarify the feedback. This may have been due to a lack of clarity in written feedback, or the greater role of the relational aspect of feedback for this cohort. These findings emphasise the importance of the contribution of past experience, context, and environment to students’ perception of feedback and consequent feedback practice [9, 18].

Many of the tensions identified in our study related to people, relationships and how people position themselves within the feedback interactions. The importance of a relationship between student and educator has been highlighted before in both medical and postgraduate students and is referred to as the educational alliance [16, 53, 54]. Our students’ view of the educator concerning their “content” expertise, investment in and presence with the student, and perceived view of the student all affected the credibility of the implicit educational alliance and, consequently the experience and perception of the student towards feedback [54]. The credibility of this alliance may be impacted by either the student, educator, or both. Hierarchy, inherent in the medical field, may overflow into feedback practice, where students place disproportionate value on the expertise of the educator and may lack confidence in themselves as seen in our study. Positioning of students in feedback has been previously reported and highlights how an awareness on the part of the educator is needed to dismantle this perceived hierarchy thereby assisting students to enact their agency [18, 55]. One way in which these hierarchies and perceived power positions can be ameliorated is through the role-modelling of “intellectual” fallibility and vulnerability on the part of the educators, which was alluded to by some students [15, 56–58].

Underpinning the alliance was what students frequently referred to as a “safe space”. While admittedly how this “safe space” is defined may be ambiguous, we sense that it was a space where they felt free to ask questions, make mistakes and be vulnerable [59]. This “safe space” can be seen as a psychological safe space, the importance of which has been highlighted in feedback literature [57, 59]. The perceived “safety” of the space, by students in our study, determined how students perceived their agency, engaged with, and experienced feedback in keeping with the literature [57, 60]. Students in this study spoke more explicitly about the role of the educator or context on the “safety”, however, the awareness of their contribution to safety may have been missed by the students [60]. The tensions we found relating to educators’ attitudes and behaviours, and the subsequent contribution to perceived safety are similar to the empirical work by Johnson (2020) [57]. The tension arising between students, community and division of labour with regards to the understanding of feedback as a continuous process, may also perpetuate the students feeling unsafe, as following a period of vulnerability on the students’ part, there was no action from the educator [57]. Trust and consequent safety may be enhanced by the educator themselves being seen as a person who is also fallible, and educators in turn, seeing every student as a person [56, 58, 61, 62]. It may be in this shared space of vulnerability that trust is built, safety is ensured, and where conversation and co-creation of learning can occur [57, 63, 64].

### Strengths and limitations

This study was conducted with a small group of final-year physiotherapy students and, therefore the understanding and experience may not apply to all students in this physiotherapy programme. Furthermore, the snowball sampling technique may have limited the breadth of experience, given that students may have invited friends with similar experiences. Given the sampling technique, it is possible that those more inclined to seek, give, receive and respond to feedback, responded. This study may have been strengthened through purposive sampling, however, the criteria for selection would need careful consideration. The conundrum, however, is what criteria should be used to purposively select students to participate and represent the possible diversity in understanding and perception of feedback. Marks may be one criterion, however, these may not necessarily represent the students’ perception and experience of feedback. It is, therefore recommend that similar studies be conducted in different contexts, at different time point in the academic programme and ensure a range of student experience.

While every effort was made to reduce the possible power dynamics, we acknowledge that this dynamic may have affected the data generated. It is clear from the findings that student perception of feedback emerges from a complex interplay of factors, admittedly not all were evaluated in this study [18, 56]. Nevertheless, the use of CHAT as an analytic lens allowed us to capture some of this complexity. Furthermore, CHAT allows us to identify actionable tensions to enhance students’ experience and perception of feedback and consequent learning. Despite the limitations, our study adds to the growing body of empirical evidence of understanding feedback practice from the students’ perspective.

## Conclusion

Students’ experience and perception of feedback was the product of the complex and dynamic interaction of various factors such as how people positioned themselves in the interaction or the complexity and enablement of contexts and environments in which they found themselves. While this study gives voice to the students in feedback practice and identifies factors that may enhance or diminish their perception of feedback and consequent learning, the complexity of feedback practice cannot be overlooked. Students expressed an understanding and recognised their agency in feedback practice, which seemed to be hindered by the tensions relating to educators and the context in our setting. Our study highlights the integral role the educator plays in nurturing students to embrace and enact their agency in feedback practice through also showing their own vulnerability. While students may have agency, how do we allow them to enact it? Perhaps it is time to shift focus from students to educators and the environments we create in optimising feedback for learning.

## Electronic supplementary material

Below is the link to the electronic supplementary material.

Supplementary Material 1

## Data Availability

No datasets were generated or analysed during the current study.

## Declarations

### Clinical trial number

N/A.
