# Supplementary material for: A qualitative exploration of feedback experience among final-year physiotherapy students using activity theory
Source: BMC Med Educ. 2025 Jan 20;25:94. doi: 10.1186/s12909-025-06635-8 (PMC11744870; doi:10.1186/s12909-025-06635-8)
Supplement: Supplementary file 1 — Supplementary Material 1 [file 12909_2025_6635_MOESM1_ESM.docx]

# Supplementary File 1

## Interview guide (probing questions)

- Tell me what your understanding is about feedback?
- Please explain the value you perceive feedback to have.
- What do you think is your role (as student) in the feedback process?
- What do you think is your responsibility in the feedback process?
- If you think back on a feedback experience, tell me about what you experienced as positive about that specific experience?
- If you think back on a feedback experience, tell me about what you experienced as negative about that specific experience?
- Has your perception on feedback changed during your years of study? Please explain why and how. (How do you think your ideas and/or experience regarding feedback have changed over the course of your studies.)
